# The Fear Factor: How Cancer Recurrence Shapes Treatment Choices About Thyroid Cancer

**DOI:** 10.1002/wjs.12583

**Published:** 2025-04-09

**Authors:** Catherine B. Jensen, Susan C. Pitt

**Affiliations:** ^1^ National Clinician Scholars Program University of Michigan Ann Arbor Michigan USA; ^2^ Department of Surgery University of Wisconsin Madison Wisconsin USA; ^3^ Department of Surgery University of Michigan Ann Arbor Michigan USA

Hampton et al. performed a discrete choice experiment in 143 patients to evaluate how the potential risk of five treatment attributes influenced hypothetical treatment choices for low‐risk differentiated thyroid cancer [1]. Although all attributes significantly influenced decision‐making, the risk of cancer recurrence had the strongest effect on treatment preference. As the risk of recurrence increased, participants' preference for surgery increased (for both hemithyroidectomy and total thyroidectomy), whereas their preference for nonoperative management with active surveillance declined. The other attributes studied included voice changes, the need for thyroid hormone supplementation, hypocalcemia requiring supplementation, and the chance of needing future thyroid surgery. The patient population studied had a mixture of diagnoses, including benign thyroid nodules (*n* = 86) and low‐risk differentiated thyroid cancer (*n* = 57); the majority had undergone thyroidectomy (*n* = 132). In the study design, participants assessed hypothetical case scenarios involving a 50‐year‐old patient with a small solitary differentiated thyroid cancer and choose between blinded treatment options that described: active surveillance, hemithyroidectomy, and total thyroidectomy. The scenarios varied each attribute's risk profile within a clinically plausible range to evaluate how each attribute's risk level affected participants’ hypothetical treatment preference.

The finding that the perceived risk of 10‐year thyroid cancer recurrence had the strongest influence on participants' treatment choice and outweighed all other treatment attributes aligns with prior work in this area. Another discrete choice study by Ahmadi et al. examined how risk perceptions influenced surgical treatment preference between hemithyroidectomy and total thyroidectomy and found that cancer recurrence risks accounted for 35% of participants' decisions [2]. A different study used constant sum scaling to demonstrate that patients with low‐risk differentiated thyroid cancer prioritize the risk of cancer recurrence over all other outcomes studied when making their actual treatment decision [3]. Patients who chose total thyroidectomy rated the risk of recurrence as significantly more important to their decision than those who chose hemithyroidectomy. However, when evaluated 9 months after surgery, the importance of recurrence decreased in those who chose total thyroidectomy, whereas the importance of energy levels significantly increased.

Another key finding of this study was participant's preference for less extensive treatment when blinded to the treatment description. Nearly, half (49%) of participants chose active surveillance over hemithyroidectomy (22%) or total thyroidectomy (29%), which does not reflect actual treatment received in many parts of the world. Although patients are more comfortable choosing less in hypothetical scenarios than in the real world, this proof‐of‐concept is important because most patients in the United States with small low‐risk thyroid cancer undergo total thyroidectomy despite its higher risk of complications. Prior work also has shown that many patients will choose active surveillance and are more receptive to less extensive treatment for thyroid cancer when options are presented in an unbiased manner [4]. These observations raise an important issue—surgeons need to consider how they describe and frame treatment options, because these details influence patients' decisions. The use of “thyroid conserving therapy” to describe hemithyroidectomy has been used in publications from Japan and represents a small change that could make a big difference in treatment choice.

This study should be interpreted in the context of how patients perceive and interpret risks, as these perceptions play a critical role in treatment decision‐making. The results highlight how altering the risk level of a treatment attribute can change patients' treatment choice. Patients and people in general often struggle with numerical risk interpretation, so‐called numeracy, which influences how they evaluate probabilities and trade‐offs in medical decisions [5]. This study systematically varied cancer recurrence risk across treatment options, presenting active surveillance with a 2%–15% risk of recurrence, hemithyroidectomy with a 2%–10% risk, and total thyroidectomy with a 1%–5% risk. Although a 5%, 10%, and 15% risk of recurrence may all be considered “low” in absolute terms, patients are more likely to fixate on relative differences—viewing 10% as twice the risk of 5% and 15% as three times the risk. This focus on small relative differences can amplify the importance and fear of cancer recurrence pushing patients toward more aggressive interventions. Similarly, small but equal absolute differences in percent change are not always perceived equally. For example, the difference between 48% and 50% may seem negligible, whereas a jump from 2% to 4% can seem disproportionately larger, despite the 2% change for both being mathematically equivalent.

Hampton et al.’s study also raises the issue of how risk perception is shaped by cultural norms, values, and belief systems, which can further complicate decision‐making. Patients and surgeons both have tendencies to be a medical “maximizer” or “minimizer,” a characteristic that influences their judgment, risk calculation, risk presentation, and treatment decision‐making. Prior studies of patients with low‐risk thyroid cancer and surgeons who treat low‐risk thyroid cancer have shown that those with maximizing tendencies favor total thyroidectomy over hemithyroidectomy [3, 6]. These results are not surprising because maximizers tend to seek the most aggressive approach to eliminate risk and presumably believe that removing “all of the cancer” equates to a more definitive cure, which will lead them to accept the higher risk of surgical complications and requirement for daily medication. Conversely, minimizers, who prioritize avoiding medical intervention, may lean toward hemithyroidectomy or active surveillance to preserve their thyroid function and avoid the need for daily medication, despite a small risk of requiring future surgery. Patient differences in information interpretation and surgeon differences in presentation reinforce the need for more intentional standardized approaches to risk communication during preference‐sensitive treatment discussions for thyroid cancer.

Hampton et al. should be congratulated for their contribution to understanding the cognitive factors that influence treatment decisions for patients with low‐risk thyroid cancer [1]. Their study provides valuable insights into how postoperative treatment outcomes shape patient preferences and highlights the significant role of cancer recurrence risk perception in decision‐making. Future research should incorporate additional important patient‐centered outcomes, such as long‐term fatigue and quality of life, to provide a more comprehensive understanding of treatment preferences. Additional research is needed to assess strategies to improve and standardize preoperative education and expectation setting to ensure that patients are well‐informed and supported in their decision‐making process.

## Author Contributions

**Catherine B Jensen:** conceptualization, project administration, writing – original draft. **Susan Pitt:** conceptualization, project administration, supervision, writing – original draft, writing – review and editing.

## Conflicts of Interest

The authors declare no conflicts of interest.

## Data Availability

The authors have nothing to report.
